# Adsorption of Chromium and Nickel Ions on Commercial Activated Carbon—An Analysis of Adsorption Kinetics and Statics

**DOI:** 10.3390/molecules28217413

**Published:** 2023-11-03

**Authors:** Joanna Lach, Ewa Okoniewska

**Affiliations:** Faculty of Infrastructure and Environment, Czestochowa University of Technology, Brzeźnicka 60a, 42-200 Częstochowa, Poland; ewa.okoniewska@pcz.pl

**Keywords:** adsorption, commercial activated carbon, nickel Ni(II), chromium Cr(III)

## Abstract

The adsorption of nickel Ni(II) and chromium Cr(III) ions on the commercial activated carbons WG-12, F-300 and ROW 08, which differ in their pore structure and the chemical nature of their surfaces, were analyzed. The nickel ions Ni^2+^ were best adsorbed on the WG-12 activated carbon, which had the largest number of carboxyl and lactone groups on the surface of the activated carbons, and the largest specific surface area. Chromium, occurring in solutions with pH = 6 in the form of Cr(OH)^2+^ and Cr(OH)_2_^+^ cations, was best adsorbed on the ROW 08 Supra activated carbon, which is characterized by the highest values of water extract. The precipitation of chromium hydroxide in the pores of the activated carbon was the mechanism responsible for the high adsorption of Cr(III) on this carbon. For the other sorbents, the amount of carboxyl and lactone groups determined the amount of Cr(III) and Ni(II) adsorption. The adsorption kinetics results were described with PFO, PSO, Elovich and intraparticle diffusion models. The highest correlation coefficients for both the Cr(III) and Ni(II) ions were obtained using the PSO model. Among the seven adsorption isotherm models, very high R^2^ values were obtained for the Toth, Temkin, Langmuir and Jovanovic models. The Cr(III) ions were removed in slightly larger quantities than the Ni(II) ions. The capacities of the monolayer q_m_ (calculated from the Langmuir isotherm) ranged from 55.85 to 63.48 mg/g for the Cr(III), and from 40.29 to 51.70 mg/g for the Ni(II) ions (pH = 6). The adsorption efficiency of Cr(III) and Ni(II) cations from natural waters with different degrees of mineralization (spring, weakly and moderately mineralized) was only a few percent lower than that from deionized water.

## 1. Introduction

The removal of heavy metals, including nickel and chromium (III), from aqueous solutions is necessary due to the frequent occurrence of these metals in sewage. These metals can be easily adsorbed by aquatic animals and directly enter human food chains, thus posing a high health risk to consumers [1,2]. Environmental protection organizations have classified heavy metal ions, including nickel and chromium, as dangerous and toxic environmental pollutants. Therefore, there is a need to treat industrial wastewater contaminated with these metal ions before discharging it into receiving bodies of water [3]. Another problem is the presence of heavy metals in drinking water [4]. According to WHO guidelines, the maximum permissible concentration of Ni(II) in drinking water is only 0.02 mg/L, while for total chromium it is 0.05 mg/L [5].

The presence of nickel in aquatic environments is due to the wide industrial use of this metal. The main industries that use it and contribute to water pollution, include galvanizing plants and plants manufacturing stainless steel, jewelry, coins, catalysts, batteries and pigments [6,7]. Nickel is one of the leading causes of respiratory problems, cardiac arrest, kidney disease damage and heart problems [8]. Nickel is listed by the Environmental Protection Agency (EPA) as a carcinogen (Group 2B). It is a nephrotoxin, an embryotoxin and a teratogenic element [9].

Chromium occurs in aquatic environments mainly as Cr(III) and Cr(VI). The trivalent form is much less toxic than the hexavalent form. Trivalent chromium is a microelement, and both its deficiency and excess may have an adverse effect on the human body and on living organisms. However, there is a real risk of the oxidation of Cr(III) into the very toxic Cr(VI), for example, during water treatment using oxidants, such as sodium hypochlorite [10,11], chlorine [12] or ozone [11,13].

Chromium is widely used in industrial processes, including metallurgy, refractory industry and chemical production (textile dyes, pigments, electroplating, leather tanning and wood preservatives) [14,15].

The scale of the problem may be evidenced by the manufacture of leather products, among other things. In Poland, in 2022, there were 2746 footwear manufacturers, 1596 bag producers and 317 entities related to leather dressing and tanning. China is the world’s leading supplier of footwear; the Polish imports of which, in 2022, amounted to 155,370,000 pairs [16]. Chromium Cr(III) is the dominant form of Cr released from the process of tanning leather. It is possible that during its production, leather may have some oxidizing capacity to convert the Cr(III) in leather into Cr(VI) [14]. Cr(VI) is 500–1000 times more toxic than Cr(III), and causes serious health effects in humans, such as skin, kidney and liver irritation, and a risk of cancer of the respiratory and gastrointestinal tracts [17,18].

Many methods have been described in the literature for effectively removing both Ni(II) and Cr(III) ions. These include precipitation, oxidation, membrane processes, coagulation, reverse osmosis, ion exchange and adsorption [19,20,21]. Various sorption materials for heavy metal removal are being investigated, e.g., iron oxide–silica nanocomposites [22] and biobased chitosan derivative [23]. Adsorption on activated carbons has many advantages; for example, it is highly effective in removing heavy metals and is a simple and relatively cheap process. It is also an easily accessible material. Moreover, when the adsorption process is used, it avoids introducing additional chemical compounds into the water. An additional advantage is the possibility of regenerating the used activated carbons and reusing them. In addition to commercial activated carbons, various waste materials are used as sorbents for Cr(III) and Ni(II) ions: watermelon rinds [24], acorn shells [25], *Litchi chinensis* seeds [26], *Citrus limetta* leaves [15], *Cordia africana* leaves [27], biosorbent from Artocarpus nobilis [28], bentonite [29], biochars from plant waste [30], waste from olive oil production [31] and many others.

This article focuses on assessing the effectiveness of removing Cr(III) and Ni(II) ions from commercial (unmodified) activated carbons used in water treatment plants. The aim of this work is to analyze and evaluate the various models of adsorption kinetics and adsorption isotherms, and to evaluate the mechanisms of this process.

## 2. Results and Discussion

### 2.1. Adsorption of Cr(III) Ions

#### 2.1.1. Adsorption Kinetics

**Figure 1:**
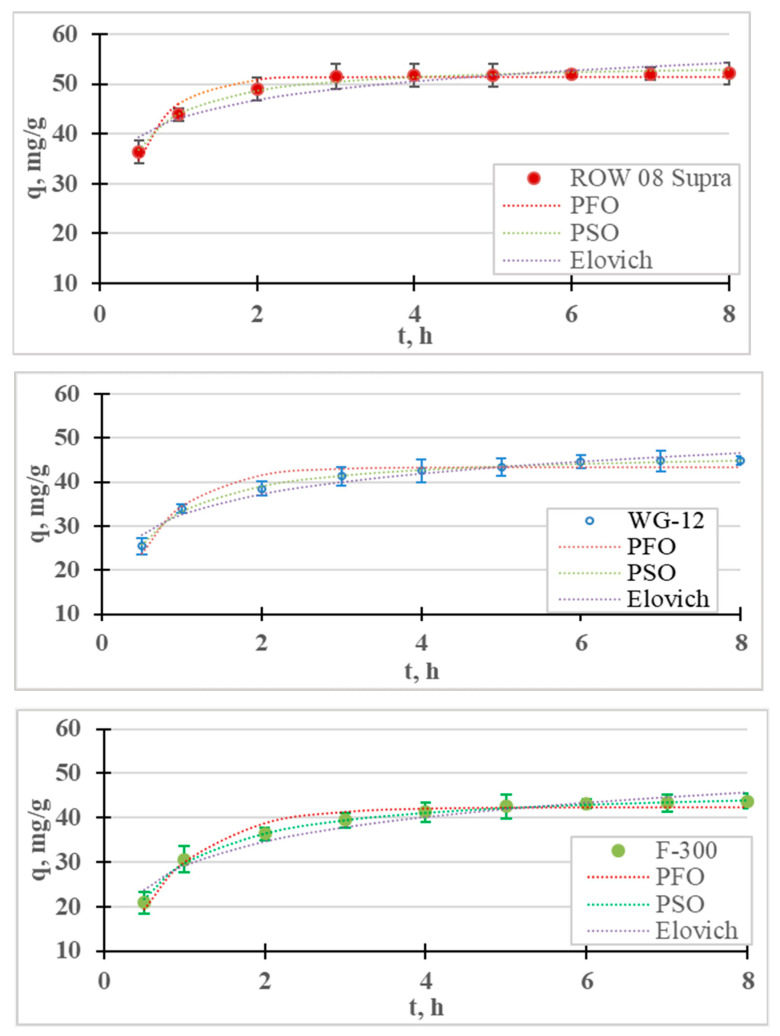
Kinetics of Cr(III) adsorption on activated carbons (PFO, PSO and Elovich models). pH = 6, C_0_ = 50 mg/L.

The results of the Cr(III) ion adsorption kinetics tests are presented in Figure 1. Adsorption kinetics measurements were carried out for 8 h, during which time equilibrium and adsorption were achieved for all the tested sorbents. It was assumed that the time for establishing adsorption equilibrium occurs when the concentration of the tested metal in the next measurement (i.e., within 1 h) changes by less than 1% of the initial concentration (0.5 mg/L). For the examined cases, the equilibrium and adsorption of Cr(III) on the ROW 08 Supra activated carbon were established after 4 h, while on the WG-12 and F-300 carbons they were established after 6 h. It was observed that the shortest equilibrium establishment time was obtained for the activated carbon absorbing the largest amounts of Cr(III) anions, and was characterized by the largest mesopores volume and the smallest micropores. A 6 h contact time for all the activated carbons within the Cr(III) solution was selected for further research. The adsorption equilibrium establishment times described by other researchers fit within a very wide range. Duran et al. [32] obtained Cr(III) adsorption equilibrium within 0.5 h, Gupta et al. [33] after 1 h, Kassahun et al. [27] after 3 h, while for Bautisto-Toledo et al. [31] it occurred only after 50 h. In most of the cases described in the literature, the time is in the range of 1–5 h [34].

The results of the adsorption kinetics studies are described with four models of adsorption kinetics. The graph contains the measurement points, with the corresponding graphs of the pseudo-first-order, pseudo-second-order and Elovich models; Table 1 shows the coefficients of these models. For all the tested sorbents, the PSO equation described the results with the highest correlation coefficient (R^2^) and the lowest error value (E). Table 2 summarizes the reports in the literature on the adsorption of Cr(III) on various sorbents. In most cases, the PSO model described this study’s results better than the PFO model. Many authors believe that the good fit of the PSO model indicates the chemical nature of adsorption, while the PFO indicates its physical nature [27,35]. According to research by, for example, Tran et al. [36] or Płaziński and Rudziński [37], the PFO and PSO models are empirical, and the adsorption mechanism cannot be determined on the basis of them. It was also found that the Lagergren PFO model can be used for adsorption systems where the adsorption time is short (up to 20–30 min) [38]. In the case of the microporous sorbents used in this research, the adsorption times were much longer. The highest Cr(III) adsorption rate (k_2_) was obtained for the ROW 08 Supra activated carbon, and the lowest for the F-300.

The Elovich model was also analyzed because, on its basis, it is possible to estimate the process rate constant α, and the coefficient β, which reflect the number of sites available for adsorption [39]. High correlation coefficients for the Elovich model (R^2^ = 0.878–0.954) indicate the significant importance of chemisorption on heterogeneous surfaces. However, chemisorption is only one of the processes responsible for the adsorption of Cr(III) ions.

**Figure 2:**
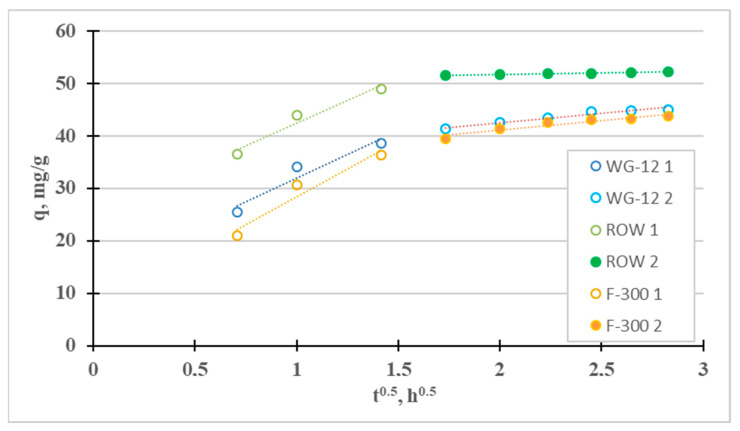
Intraparticle diffusion curves for the adsorption of Cr(III).

The fourth adsorption kinetics model considered is the intraparticle diffusion model (Weber–Morris). It was used to identify the mass transfer mechanism. There are four steps involved in the mass transport process during adsorption. The first stage—the so-called “mass transport” (transport in the solution phase)—is very fast; therefore, this stage can be considered irrelevant, for example, when considering the time to establish adsorption equilibrium. The next stage—the so-called “film diffusion”—which involves the transport of mass from the liquid phase to the outer surface, is much slower. The third stage—the so-called “intraparticle diffusion”—involves the diffusion of adsorbate molecules from outside the adsorbent into the pores of the adsorbent, along the surface of the pore walls. It is also a slow process. The final stage is adsorption, which occurs very quickly. Figure 2 shows the IPD graph divided into the two slowest phases. For the tested sorbents, the third stage is much slower than the second, so diffusion in the pores (k_p2_ < k_p1_) controls the sorption of Cr(III). Intraparticle diffusion is not the single rate-limiting step during adsorption because the q_t_ curve from t^1/2^ does not pass through the origin of coordinates [40]. A similar effect from intraparticle diffusion on the adsorption of Cr(III) ions was obtained by Lesaoana et al. [35] and Duran et al. [32].

**Table 1:** Constants of Cr(III) adsorption kinetics equations: the pseudo-first-order, pseudo-second-order, intraparticle diffusion (Weber–Morris) and Elovich models.

Model	Parameter	WG-12	ROW	F-300
Pseudo-first order $\frac{dq_{t}}{dt}=k_{1}(q_{e}-q_{t})$	q_max_ (mg/g)	43.32	51.42	42.32
	k_1_ (h^−1^)	1.588	2.254	1.236
	R^2^	0.935	0.947	0.967
	E, %	3.51	2.05	3.29
Pseudo-second order $\frac{dq_{t}}{dt}=k_{2}{\left(q_{e}-q_{t}\right)}^{2}$	q_max_ (mg/g)	49.30	54.36	47.27
	k_2_ (h^−1^)	0.050	0.077	0.036
	R^2^	0.996	0.989	0.996
	E, %	0.84	0.87	1.05
Elovich $\frac{dq_{t}}{dt}=\alpha e^{-\beta q_{t}}$	α, mg/(g·h)	821	15929	298
	β, g/mg	0.148	0.186	0.125.
	R^2^	0.954	0.878	0.950
	E, %	3.12	3.33	4,43
$q_{t}=k_{p}t^{0.5}+C$ Intraparticle diffusion model (Weber–Morris)	k_p1_, mg/(g·h^0.5^)	18.02	17.39	21.32
	C_1_, mg/g	13.92	25.00	7.11
	R^2^	0.926	0.958	0.940
	k_p2_, mg/(g·h^0.5^)	3.50	0.62	3.72
	C_2_, mg/g	35.52	50.42	33.665
	R^2^	0.938	0.993	0.919

q_m_ (mg/g)—the amount of solute adsorbed at equilibrium; q_t_ (mg/g)—the amount of solvent adsorbed at time t; k_1_/k_2_ (h^−1^)—the rate constant for the pseudo-first-order/pseudo-second-order kinetic model; α (mg/(g·h))—the initial adsorption rate; β (g/mg)—reflects the number of sites available for adsorption; k_p_ (mg/(g·h^0.5^))—the intraparticle diffusion rate constant; C (mg/g)—the intercept, which is a constant related to the thickness of the boundary layer; t^0.5^—half life time.

**Table 2:** Comparison of Cr(III) adsorption results based on the reports in the literature.

Activated Carbon	Max. q_m_, mg/g	Adsorption Isotherms Equations Tested	The Equations of Adsorption Kinetics Studied	Ref.
From corncob waste	84.64	L > F	PSO > PFO	[41]
From macadamia	69.61	F > L	PSO > PFO	[35]
From wood	185.18	L > F > T > D–R	PSO > PFO	[40]
From leather industry solid wastes	220	T > F > L	PSO > Elovich > IDP > PFO	[42]
From olive waste	12.46	L = T > F	PSO > PFO	[43]
From fish scale	18.34	L > F	PSO > PFO	[44]
From cordia africana	0.011	F > L	PFO > PSO	[27]

#### 2.1.2. Adsorption Statics

Adsorption tests under static conditions were carried out for 6 h. This amount of time, based on adsorption kinetics studies, was sufficient to establish equilibrium on all of the three tested activated carbons. Measurements were carried out for solutions with a pH = 6, in which chromium hydroxide precipitation had not yet occurred, and in which Cr(III) adsorption was at a maximum, according to many studies [32,33,40]. Measurements were carried out for solutions with an initial concentration of 10–100 mg/L. Similar concentrations have often been considered by other researchers [33,40].

**Figure 3:**
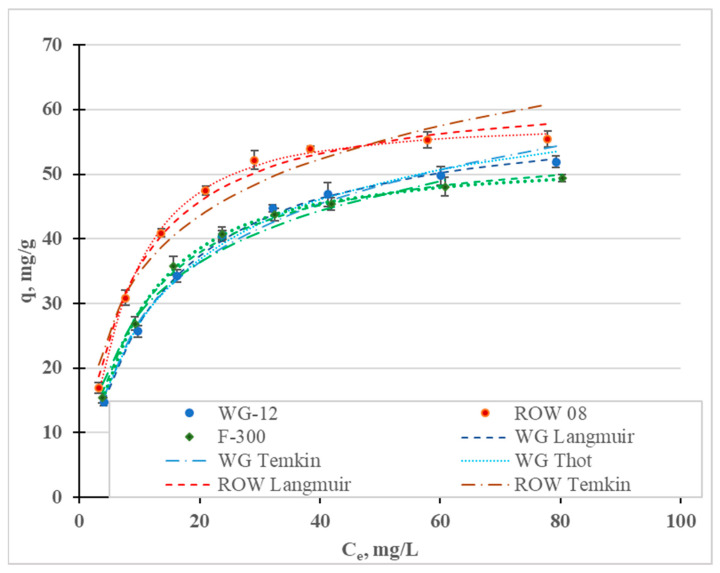
Cr(III) adsorption isotherms for the activated carbons.

**Figure 4:**
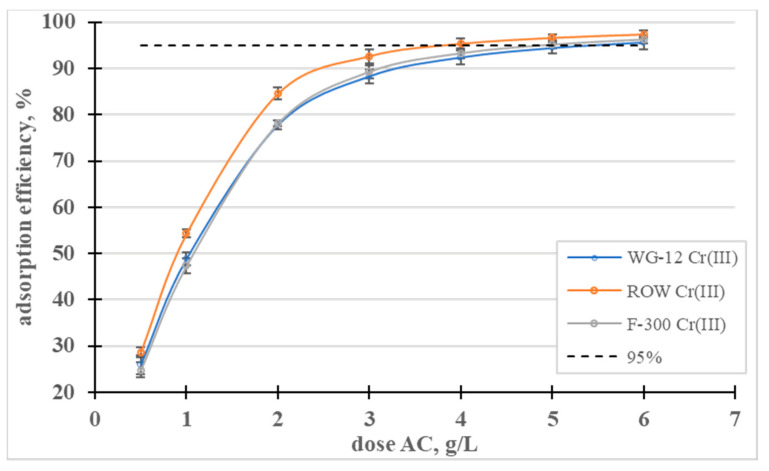
Influence of the activated carbon dose on the efficiency of Cr(III) removal.

Figure 3 shows the Cr(III) adsorption isotherms; Table 3 shows the calculated coefficients of the two-parameter Langmuir (L), Freundlich (F), Temkin (T), Javanovic (J) and Halsey (H) isotherm models, and the three-parameter Redlich–Peterson (R-P) and Toth (To) models. These isotherms differ in their assumptions regarding, among other things, mono- or multilayer adsorption, adsorption mechanisms (chemical or physical), hetero- or homogeneity of the surface and the interactions, or lack thereof, between the adsorbed adsorbate molecules [45]. In Figure 4, in addition to the measurement points, are graphs of the isotherm models for which the highest R^2^ and lowest E were obtained: the Temkin (R^2^ 0.995–0.999), Toth (R^2^ 0.992–0.999) and Langmuir (R^2^ 0.988–0.997). High correlation coefficients were also obtained for the Jovanovic model (R^2^ 0.990–0.997).

Fitting the models to the test results is not a basis for determining the nature of adsorption [36]. The four models that best describe Cr(III) adsorption differ in their basic assumptions. The Temkin model assumes chemical adsorption, the Toth and Jovanovic models assume physical adsorption and the Langmuir model refers to both chemical and physical adsorption. All these isotherms assume single-layer sorption. The only model considered that assumes multilayer sorption (Halsey) was characterized by the lowest correlation coefficients.

When analyzing the literature on the adsorption of heavy metals, including Cr(III), it can be seen that the Langmuir and Freundlich equations (Table 3) are the dominant models (due to the frequency of their use). In this study, higher values of the correlation coefficients were obtained for the Langmuir model than for the Freundlich model. Reports in the literature on the fit of specific models to the results of Cr(III) adsorption tests are not clear (Table 3). Depending on the sorbents used, different models are characterized by having the highest correlation coefficients.

The favorable adsorption of Cr(III) is demonstrated by the separation coefficients R_L_ = 0.83–0.98 (Table 3), calculated from the Langmuir isotherm equation, which are in the range 0–1. A similar conclusion results from the analyzed values of the coefficient, n, from the Freundlich isotherm. The n values are 2.9–3.6, i.e., in the range of 1–10. The 1/n values indicate the degree of heterogeneity of the active sites on the surface of the sorbents. If the values of 1/n are close to zero, the surface is homogeneous; if the values are close to 1, they indicates a large heterogeneity of the adsorbent surface. In the case of the studies discussed above, the 1/n values are in the range of 0.28–0.34, which indicates an average heterogeneity of the specific surface area of the carbon sorbents used in the studies.

The Langmuir, Toth and Jovanovic models determine the monolayer capacity, q_m_ (Table 3). The q_m_ value calculated from the Langmuir isotherm ranges from 55.85 mg/g for the F-300 carbon to 63.49 mg/g for the ROW 08 Supra. The q_m_ values calculated from the Toth isotherm are similar (from 52.42 mg/g to 68.90 mg/g), while those from the Jovanovic are slightly lower (47.65–54.94). Regardless of the model used, Cr(III) adsorption is the highest on the ROW 08 Supra activated carbon and the lowest on the F-300. The obtained values for sorption capacity during the adsorption of Cr(III) on commercial activated carbons are average, as compared to the capacities obtained by other researchers, which are listed in Table 3. Taking these capacities into account, the activated carbons can be ranked in the following order: ROW 08 Supra > WG-12 > F-300. However, the differences between these carbons are not significant. These carbons are also characterized by similar pH_ZC_ values (6.4–6.6) (Table 4). Under the testing conditions (pH = 6), the surface of the activated carbons has a slightly positive charge, which does not favor the electrostatic attraction of Cr(III) cations. According to Bautista-Toledo et al. [31], the adsorption of Cr(III) is specific, and takes place mainly on the carboxyl and lactone groups on the surface of activated carbon. At pH = 6, the carboxyl groups with pKa = 3–6 and, to a lesser extent, the lactone groups with pKa = 7–9, are in an ionized form and are responsible for the adsorption of Cr(III) cations. Research by Jimenez-Paz et al. [42] and Dash et al. [43] proves that—in addition to carboxyl and lactone groups—amino groups also influence the adsorption of Cr(III). Moreover, according to the research by Kaźmierczak et al. [30] conducted on biosorbents, Cr(III) ions can form chelate complexes with surface groups.

Taking into account the chemical structure of the surface, the following adsorption mechanisms can be proposed:

- On amphoteric and basic activated carbons:

AC: +Cr(OH)^2+^ → AC: Cr(OH)^2+^ (Cπ-cation interactions) (1)

AC: +Cr(OH)_2_^+^ → AC: Cr(OH)_2_^+^ (Cπ-cation interactions) (2)

- On “acidic” activated carbon:

AC–COOH + Cr(OH)_2_^+^ + H_2_O → AC–COO Cr(OH)_2_ + H_3_O^+^ (3)

(AC–COOH)_2_ + Cr(OH)^2+^ + 2H_2_O → (AC–COO)_2_Cr(OH) + 2H_3_O^+^ (4)

In general, the FTIR diagrams can be divided into four ranges: 4000–2000, 2000–1300, 1300–900 and 900–600 cm^−1^ [46]. The first range (4000–2000 cm^−1^) is characterized primarily by free O-H, H-bonded OH, adsorbed H_2_O and symmetric and asymmetric stretching in CH-, CH_2_- or CH_3_- bonds. From the point of view of adsorption, the range of 2000–1300 cm^−1^ is the most interesting. It characterizes the most important oxygen functional groups containing C=O and N–O structures in carbonyls, lactones, aldehydes and carboxyl radicals. The third range is characterized by single C–O bonds occurring in ethers, esters, phenols and hydroxyl groups. Shoulder bands at lower wave numbers (830, 760, 670 and 600 cm^−1^) might be associated with the out-of-plane bending modes of C–H, as in benzene.

**Figure 5:**
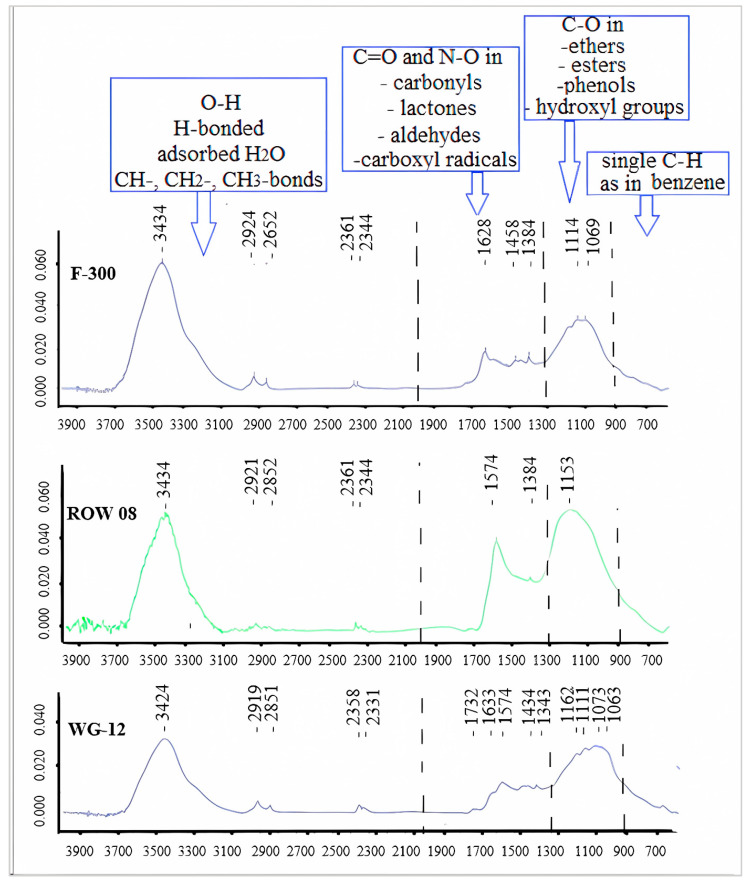
FTIR spectra for the tested activated carbons.

When analyzing the number of carboxyl and lactone groups using the Boehm method, a small number of them can be found, especially on the ROW 08 Supra carbon, which adsorbed the largest amount of Cr(III) cations. A small number of these groups were confirmed using the FTIR spectrum (Figure 5). An intense band corresponding to secondary amines can be observed on the ROW 08 Supra carbon, which adsorbed the largest amount of Cr(III) cations (1574 nm). This carbon also has the largest number of basic groups (Boehm’s method—Table 5). The F-300 activated carbon, which adsorbed the smallest amount of Cr(III) ions, is characterized by an average amount of carboxyl groups assessed using the Boehm method. Analyzing the FTIR spectra, one can notice bands of low intensity responsible for the primary amines (1628 nm).

The inability to directly link the amounts of adsorbed Cr(III) cations and acidic groups (especially the carboxyl groups) proves the complex nature of the phenomenon under study. The porous structure also has an impact. The WG-12 activated carbon with the largest amount of acidic groups (including carboxyl and lactone groups) is characterized by the largest specific surface area and, therefore, the largest share of micropores in their total volume. Some of the functional groups are located in the smallest micropores, and are inaccessible to the hydrated Cr(III) cations. At the same time, the activated carbon ROW 08, which sorbs the largest amount of Cr(III), has the largest mesopore volume, which, in this case, should be considered favorable. There is also the possibility of the precipitation of chromium hydroxide in the micropores in the presence of the remains of unwashed alkaline ash. In the case of the ROW 08 Supra activated carbon, the pH of the water extract is clearly higher than that of the other activated carbons (8.6 for the ROW 08 Supra and 6.7–6.8 for the other sorbents). The end effect is the resultant of several different processes.

The effect of the dose on the adsorption efficiency was also analyzed (Figure 4). The lowest of the analyzed doses, 0.5 g/L, allowed for the removal of less than 30% of the Cr(III) ions from the solution with a concentration of 100 mg/L, on all the activated carbons. A dose of 4 g/L allowed for the removal of 95% in the case of the ROW 08 Supra activated carbon but, for the other sorbents, the dose had to be 6 g/L to achieve the same effect.

**Table 4:** Physical and adsorption properties of activated carbons used in the research [45,47,48].

Parameter	Unit	Activated Carbon		
		WG-12	ROW 08	F-300
Bulk density (PN-EN 12915)	g/dm^3^	424 ± 27	381 ± 16	542 ± 36
Surface area (BET)	m^2^/g	1098 ± 38	897 ± 30	847 ± 29
pH of the water extract (PN-82/C-97555)	-	6.7	8.6	6.8
Pore structure V_total_ V_macr_ V_mezo._ V_micr._	cm^3^/g cm^3^/g cm^3^/g cm^3^/g	0.990 0.400 0.066 0.524	1.135 0.246 0.453 0.436	0.987 0.217 0.294 0.476
Iodine adsorption, LI (PN-EN 12902)	mg/g	1050	1096	1065
Methylene blue number, LM (PN-82/C-97555.03)	cm^3^	30	30	28
Grain composition—sieve analysis (PN-EN 12902) >2.0 mm 2.0 ÷ 1.5 mm 1.5 ÷ 1.0 mm 1.0 ÷ 0.5 mm <0.5 mm	%	4.9 57.4 34.3 2.2 1.0	36.4 41.2 21.7 0.3 0.1	31.4 23.4 30.2 10.2 4.6

**Table 5:** Isoelectric point and chemical structure of the surface of activated carbons determined using the Boehm method [47].

Activated Carbon		WG-12	ROW 08	F-300
Isoelectric Point, pH_PZC_	-	6.4	6.5	6.6
Acidic groups (Boehm method) Carboxylic groups -COOH Lactonic group -COO- Phenolic groups -OH Carbonyl groups =CO Basic groups/sites	mmol/g	0.586 0.182 0.209 0.110 0.085 0.467	0.434 0.063 0.120 0.409 0.021 0.592	0.544 0.138 0.048 0.316 0.060 0.512

### 2.2. Adsorption of Ni(II) Ions

#### 2.2.1. Adsorption Kinetics

The studies on the adsorption kinetics of Ni(II) ions were carried out in a similar manner as for the Cr(III) ions. Similarly, it was assumed that the adsorption equilibrium occurs when the concentration difference between two consecutive measurements is less than 1% of the initial concentration. This assumption results from the need to limit the laboratory tests, even though the actual adsorption on microporous materials is often a very long process. Kim et al. [49] did not achieve an equilibrium state, even after 75 days, in their studies of the adsorption of methyl violet on granulated activated carbon. Another problem is the possibility of the desorption of pollutants if the adsorbate is in contact with the adsorbent for too long [50]. That is why, among other reasons, kinetics studies are so important—they determine the optimal adsorption time for further measurements.

**Figure 6:**
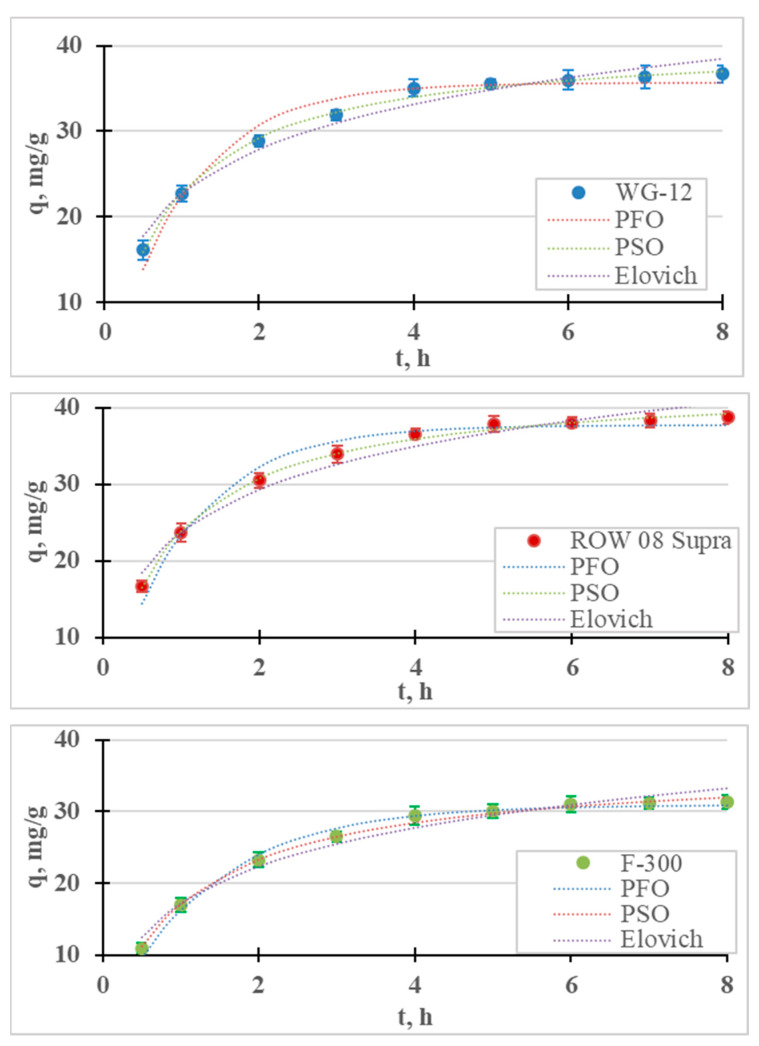
Ni(II) adsorption kinetics on activated carbons (PFO, PSO and Elovich models). pH = 6, C_0_ = 50 mg/L.

The kinetic results and calculated kinetic models of Ni(II) ion adsorption are presented in Figure 6 and Table 6. As in the case of Cr(III) adsorption, the removal of Ni(II) cations is fastest in the first stage of the process. After 1 h of the process, the adsorption efficiency of the nickel ions ranged from 13.6% (F-300) to 18.9% (ROW 08 Supra), while after 8 h these efficiencies ranged from 25.1% (F-300) to 31.0% (ROW 08 Supra). After the first hour of the process, over a 50% removal of the nickel ions was achieved compared to the total adsorption. The adsorption efficiencies obtained under the test conditions are not very high, which is due to the high initial concentration (50 mg/L) and low dose of the sorbent (0.4 g/L). Khedri et al. [15], using activated carbon from Mespilus germanica Lea, found this dose to be optimal at a similar concentration of Ni(II) ions (60 mg/L).

Differences in the final concentrations of less than 0.5 mg/L between the two tests were obtained after 5 h for the WG-12 and after 6 h for the ROW 08 Supra F-300.

The time to establish adsorption equilibrium was the shortest for the WG-12 carbon, which resulted from the highest adsorption rate calculated from the PSO kinetics equation (k_2_ = 0.031 h^−1^). The ROW 08 and F-300 had the same time to establish adsorption equilibrium, despite the fact that they characterized by different adsorption rate coefficients. The ROW 08 Supra was characterized by a higher k_2_ coefficient (0.029 h^−1^) than the F-300 (0.024 h^−1^), but it also adsorbed a larger amount of Ni(II) ions. Due to the obtained test results, further measurements were performed for a 6 h contact time. The adsorption equilibrium establishment time obtained by other researchers varies widely, which is mainly a result of the sorbent used, but also the process conditions (for example, mixing speeds) [36]. Depending on the sorbent used, other researchers have obtained very different times for establishing Ni(II) adsorption equilibrium: for example, 20 min. [51], 3 h [52], 4 h [53] and even 25 h [54].

The test results are described with four models of adsorption kinetics (Table 6). All the models with high R^2^ values (>0.88) describe the obtained research results. Among the analyzed models, the PSO equation describes the results with the highest correlation coefficient (R^2^) and the lowest error value (E). Table 7 summarizes the reports from the literature on the adsorption of Ni(II) on various sorbents. For all the research results presented in the summary, the PSO model describes the results better than the other analyzed models. The Lagergren PFO model describes the process better when the adsorption time is very short. This situation did not occur in the presented studies because the time for establishing the adsorption equilibrium was much longer than 30 min. The Elovich model is also characterized by high values of the correlation coefficient (R^2^ = 0.968–0.971), which may indicate the importance of chemisorption on a heterogeneous surface.

**Figure 7:**
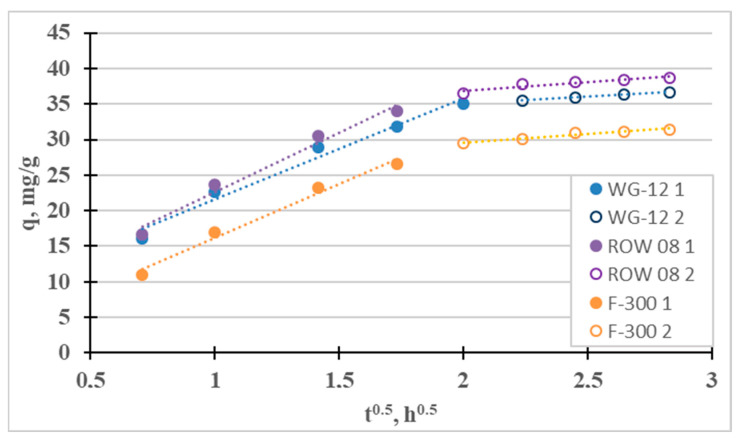
Intraparticle diffusion curves for the adsorption of Ni(II).

The fourth adsorption kinetics model considered is the intraparticle diffusion model (Weber–Morris), which can be used to determine the mass transfer mechanism (Figure 7). Two stages can be distinguished for the tested sorbents: the quite fast “film diffusion” and the much slower intraparticle diffusion. The third stage is much slower than the second one (k_p2_ < k_p1_) for all three tested active carbons. Diffusion in the pores, in such a case, controls Ni(II) sorption (the q_t_ curve from t^1/2^ does not pass through the origin of coordinates 0.0, i.e., C > 0). Similar results were obtained, for example, by El-Sadaawy et al. [3] and Manjuladev et al. [52].

#### 2.2.2. Adsorption Statics

**Figure 8:**
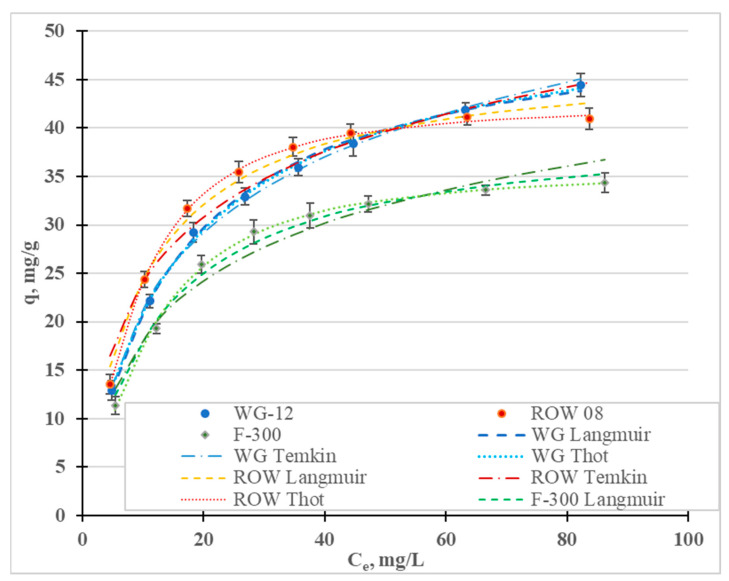
Ni(II) adsorption isotherms (pH = 2; C_0_ = 10–100 mg/L, t = 6 h).

Figure 8 shows the adsorption isotherms of the Ni(II) ions, and Table 8 shows the coefficients of the Freundlich, Langmuir, Temkin, Jovanovic, Halsey, Redlich–Peterson and Toth models. The highest values of the R^2^ coefficient and the lowest values of the E errors were obtained for the Toth (R^2^ = 0.998–0.999, E = 0.52–1.39%), Temkin (R^2^ = 0.996–0.999, E = 0.13–0.54%), Langmuir (R^2^ = 0.986–0.998, E = 1.15–3.58%) and Jovanovic (R^2^ = 0.978–02997, E = 1.42–5.04) models. Graphs for the first three models are shown in Figure 8. The Freundlich and Redlich–Peterson models, which are compilations of the Freundlich and Langmuir isotherms, are also characterized by high R^2^ values. Among the considered isotherms, the lowest R^2^ values and the highest E error were obtained for the Halsey isotherm, which assumes multilayer sorption. The remaining models assume single-layer sorption. Moreover, the Toth and Temkin isotherms, characterized by the highest R^2^ value, assume the heterogeneity of the surface of the activated carbons. However, these two models describe two different types of adsorption: Toth—physical adsorption and Temkin—chemical adsorption. However, when taking into account only adsorption isotherms or kinetics equations, the adsorption mechanism cannot be assessed. It is necessary to supplement this information and to verify, using other analytical techniques, the thesis of whether the adsorption is chemical or physical in nature.

The research results have most often been described in the literature using the Langmuir and Freundlich models. In most cases, the Langmuir model is characterized by a higher correlation coefficient than the Freundlich model (Table 7). In the research presented here, the highest correlation coefficient for all three tested carbons was obtained for the Toth model. Depending on the activated carbon, the models are ranked based on the correlation coefficient as follows: WG-12—To = T > L > J; ROW 08 Supra—To > T > J > L; F-300—To > T = J > L. Despite some differences between these series, it was observed for two of the activated carbons that the To, T and J models described the nickel ion adsorption process better than the L model and, in the case of the activated carbon WG-12, only the To and T models had higher correlation coefficients than the Langmuir model. The differences between the correlation coefficients for the isotherms discussed (To, T, J and L) are small. Despite this, it should always be considered whether the Langmuir and Freundlich models most often used for specific sorbents, sorbates and process conditions are the best choice when modeling adsorption statics.

The Langmuir, Toth and Jovanovic models determine the monolayer capacity, q_m_. The q_m_ value calculated from the Langmuir isotherm ranges from 40.29 mg/g for F-300 to 51.70 mg/g for WG-12. The q_m_ values calculated from the Toth isotherm are very similar: from 35.64 mg/g (F-300) to 54.67 mg/g (WG-12). Acidis 6 shows the sorption capacities of the various activated carbons. It can be concluded that the sorption capacities of the commercial activated carbons obtained in the tests are characterized by an average capability for removing nickel ions.

Analyzing the R_L_ values calculated from the Langmuir isotherm, and n from the Freundlich isotherm, it can be concluded that the adsorption of Ni(II) anions is good on all the activated carbons used in this study (the RL value ranges from 0 to 1; n ranges from 1 to 10).

Taking these capacities into account, the activated carbons can be ranked in the following order: WG-12 > ROW 08 Supra > F-300. This ranking coincides with the ranking according to the total amount of acid oxides, and is due to the amount of carboxylic oxides on the tested activated carbons (Table 5). The ranking of the carbons according to the amount of sorption of nickel ions is the same as the ranking according to the volume of micropores, which are the main adsorption sites for nickel ions, and for which the ionic radius is small and amounts to 0.069 nm [63]. Moreover, the carbon that absorbs the most Ni(II) ions has the largest specific surface area (Table 9), but Ni(II), which absorbs the fewest ions, does not have the smallest specific surface area. This parameter is, therefore, not decisive for the amount of sorption of the ions in question.

Adsorption was carried out using a solution with pH = 6. This is a value that is considered optimal by many authors [56,58], but guarantees that the nickel ion will not precipitate in the solution [64].

The most likely mechanism for the adsorption of Ni^2+^ ions is an electrostatic attraction between the nickel cation and the deprotonated carboxyl groups (6), and an attachment by an ionic bond to the C-O groups [2,55].

2AC–COOH + Ni^2+^ + H_2_O → (AC–O–COO^−^)Ni^2+^+ 2H^+^ (5)

2AC–COOH + Ni^2+^ + H_2_O → (AC-COO)Ni +2H^+^ (6)

Sheng et al. [65] found that the adsorption of heavy metal ions, including nickel, is influenced not only by the carboxyl groups, but also by the ether, alcoholic and amino groups.

**Figure 9:**
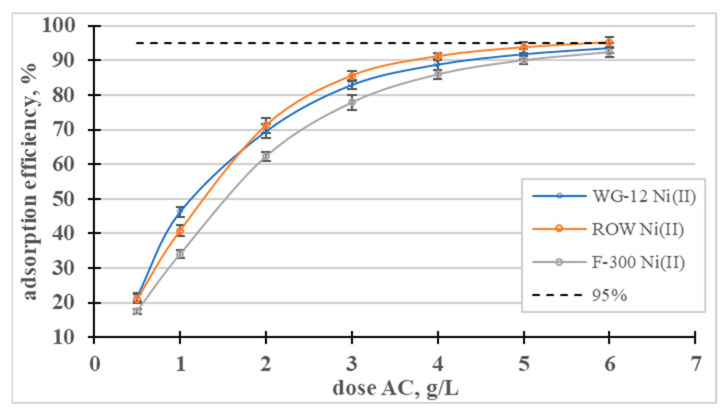
Influence of the dose of activated carbons on the adsorption efficiency of Ni(II) ions.

The influence of the dose of activated carbons on the adsorption efficiency of Ni(II) ions was analyzed (Figure 9). In a dose range from 0.5 to 3, the dose-dependent adsorption efficiency curves rise sharply and then gradually flatten out. The lowest dose (0.5 g/L) resulted in the removal of approximately 20% of the nickel ions, and a dose of 5 g/L resulted in the removal of over 90% of the Ni(II) in all the tested sorbents.

### 2.3. Adsorption of Tested Ions from Natural Waters

The influence of the composition of natural waters on the adsorption efficiency of Cr(III) and Ni(II) ions was assessed (Table 9). This study was conducted on waters whose chemical composition varied widely. Four spring waters, and four with different degrees of mineralization, were used in this study. These waters contained competing cations of Ca^2+^, Mg^2+^, Na^+^ and K^+^. Their concentrations (especially of calcium cations) were many times higher than those of the Cr(III) or Ni(II) cations. The calculated efficiencies were slightly lower when the adsorption process occurred in waters with low mineralization (spring waters). It was observed that the higher the degree of mineralization (a higher amount of competing cations), the lower the adsorption efficiency. However, the differences in effectiveness were small (a few percent). Only in the case of water with a high degree of mineralization, for which the concentration of total cations was high (more than 390 mg/L, including divalent cations of more than 220 mg/L), was the efficiency in comparison to the sorption from deionized water more than 13% lower for the Cr(III) ions and 11% for the Ni(II) cations.

### 2.4. Mechanism of Adsorption of Cr(III) and Ni(II) Cations

The ions under study occur in solutions at pH = 6 in slightly different forms. Nickel occurs as the Ni^2+^ cation, and Cr(III) as the hydrated monovalent or divalent Cr(OH)^2+^ and Cr(OH)^2+^ cations. Another difference relevant to the present study is the formation of insoluble hydroxides. Chromium(III) forms a hydroxide in solutions above pH 6.3, and nickel in solutions above pH 8.5. Both Cr(III) and Ni(II) cations are adsorbed according to the mechanisms shown in Equations (1)–(6). At the tested pH = 6, the carbons have a slightly positive charge (pHPZC from 6.6 to 6.7). The carboxyl groups (pKa = 3 ÷ 6) and, to a lesser extent, the lactone groups (pKa 7 ÷ 9), are in an ionized form. There is an electrostatic attraction between the Cr(III) or Ni(II) ions and the deprotonated carboxyl/lactone groups, and an attachment to the C-O group by an ionic bond. Nickel adsorption is determined by the presence of these groups on the carbon surface. The best-adsorbing nickel ion activated carbon, WG-12, has the most carboxyl as well as lactone groups, as measured using the Bohemian method. The least Ni(II) adsorbing activated carbon has the fewest carboxyl and lactone groups. The measurement of the amount of groupings using the Bohemian method correlates with the amount of oxygen as determined using the SEM/EDS method (Table 10). The highest amount of oxygen was determined to be on the surface of the activated carbon WG-12, which has the highest amount of acidic oxygen groupings, as determined using the Bohemian method. The smallest amount of oxygen on the surface was found for the activated carbon ROW 08 Supra, which also has the smallest amount of oxygen groupings, according to the Bohemian method. The electrostatic attraction and ion exchange mechanism are responsible for the sorption of Ni(II) cations. For the adsorption of Cr(III) ions, adsorption occurs best on the ROW 08 Supra activated carbon, despite the low amount of carboxyl and lactone groupings. In this case, the additional mechanism is the precipitation of chromium hydroxides in the pores due to the high pH of the aqueous extract (pH 8.6). The high pH of the aqueous extract for the ROW 08 Supra carbon is due to the presence of calcium in the pores, which was determined using SEM/EDS (Table 10). For the other two activated carbons, for which the pH of the aqueous extract is 6.7 and 6.8, Cr(III) adsorption depends on the presence of carboxyl and lactone groups, similarly to nickel adsorption.

On all the activated carbons analyzed, the adsorption of chromium ions occurs in greater amounts than that of nickel ions. Since nickel occurs as a divalent ion, and chromium as a divalent and monovalent ion, the same number of functional groups can adsorb more chromium ions. Furthermore, in the case of the ROW 08 Supra activated carbon, an additional adsorption mechanism is the precipitation of chromium, in the form of hydroxides.

The effect of the presence of carboxyl and lactone groups on the adsorption of Cr(III) and Ni(II) ions was also found by other researchers [31,42,55,66].

## 3. Materials and Methods

### 3.1. Adsorbents Used in this Research

The tests were carried out on three commercial carbon sorbents: Filtrasorb 300 (Chemviron, Carbon, Moon Township, PA, USA), WG-12 (Gryfskand, Gryfino, Poland) and ROW 08 Supra (NORIT, Auderghem, Belgium). These are granulated carbon sorbents used in water treatment plants. Their characteristics are presented in Table 4 and Table 5. Moreover, in order to characterize the chemical structure of the surface of the activated carbons, FTIR transmission spectra were determined (Figure 5). The FTIR spectra were acquired using a Perkin-Elmer Spectrum 2000 FTIR spectrometer (Perkin-Elmer, Waltham, MA, USA). The measurements were carried out in the wave number range 4000–400 cm^−1^, at a scanning speed of 0.2 cm/s. For the FTIR measurements, pellets were made from a mixture of activated carbons and KBr (1:300).

In order to investigate the surface topography and texture of the investigated activated carbons, SEM-EDS studies were performed. Measurements were made using a LEO Electron Microscopy scanning electron microscope (1430 VP) (Thornwood, NY, USA). Three replicates were performed for each sample. A 500 × 400 micrometer area was analyzed. The surface penetration depth was approximately 5 micrometers. Table 10 shows the results of the elemental composition analysis of the surfaces of the activated carbons tested.

### 3.2. Solutions of Heavy Metals Used in this Research

Metal nitrates were used to prepare the Ni and Cr(III) solutions: Ni(NO_3_)_2_ · 6H_2_O and Cr(NO_3_)_2_ · 9 H_2_O from Sigma-Aldrich. Stock solutions with a concentration of 1000 mg/L were made by dissolving the compounds in deionized water. Measurements were carried out for solutions with pH = 6 ± 0.2, in which the tested metals were dissolved. At these pH values, nickel exists in the form of the divalent ion Ni^2+^ [64]. According to the research by Bautista-Toledo et al. [29], trivalent chromium occurs in a solution with pH = 6 in various forms of Cr(OH)^2+^ and Cr(OH)^2+^ hydrocomplexes. According to Fonseca-Correa et al. [41], there are more forms of hydrocomplexes in a solution with pH = 6: Cr(OH)^2+^ (40%), Cr(OH)_2_^+^ (35%) and Cr_3_(OH)_4_^5+^ (25%).

Adsorption measurements of the Ni(II) and Cr(III) from natural waters were carried out. Eight bottled waters were used, which were enriched with Cr(III) and Ni(II) ions. Waters differing in their degree of mineralization were used for this study: 1 highly mineralized, 2 medium mineralized, 1 low mineralized and 4 spring waters. The characteristics of the waters are presented in Table 9. The concentration of the tested Cr(III) or Ni(II) ions was 10 mg/L, pH = 6. The effect of the composition of the natural waters on the adsorption of the tested ions was assessed by the adsorption efficiency.

The contents of Ni(II) and Cr(III) were determined using an inductively coupled plasma atomic emission spectrometer.

### 3.3. Adsorption Kinetics and Statics Studies

The adsorption tests were carried out at room temperature, according to the scheme presented in Table 11. The solution of heavy metals with activated carbon was mixed on a mechanical shaker at a speed of 160 rpm. Based on adsorption kinetics measurements, a 6 h contact time of the solution with activated carbon was assumed for all the heavy metals.

The following kinetic models were used to describe the adsorption kinetics: the pseudo-first-order, pseudo-second-order, Elovich and intraparticle diffusion models (Weber–Morris) [67]. The following models were used to describe the adsorption statics: Freundlich (F), Langmuir (L), Temkin (T), Javanovic (J), Halsey (H), Redlich–Peterson (R-P) and Toth (To) [68]. The constants for these models were determined from non-linear functions (non-linear regression method) using the Excel Solver add-in. The usefulness of the adsorption kinetics and statics models for describing the research results were assessed based on the correlation coefficient (R^2^) and the error (E) determined using Formula (7).

(7) $$\%E=\frac{100}{N}\sum_{i=1}^{N}\left|\frac{q_{e,exp}-q_{e,cal}}{q_{e,exp}}\right|$$

where:

N—number of measurement points;

q_e,exp_—adsorption capacity resulting from the measurements;

q_e,cal_—adsorption capacity calculated using a specific model.

## 4. Conclusions

The heavy metals analyzed during the tests occur in various forms in a solution with pH = 6. Under these conditions, trivalent chromium occurs in the form of Cr(OH)^2+^ and Cr(OH)_2_^+^ cations, and nickel in the form of divalent Ni^2+^ cations. These ions differ not only in molar mass and size, but also in valence. Comparing the amounts of adsorption of these ions, it was found that higher adsorption capacities were obtained in the case of the adsorption of Cr(III) ions, despite the fact that chromium has a slightly smaller molar mass and whole ions are adsorbed. The reason for this was the presence of monovalent Cr(OH)_2_^+^ ions, which use only one functional group on the surface of active carbons (e.g., a carboxyl group). Moreover, chromium hydroxide occurs already at pH = 6.3, while nickel hydroxide occurs at pH > 8. In the case of Cr(III) adsorption, there was this additional mechanism—the precipitation of Cr(OH)3 in the micropores. The capacities of the q_m_ monolayer (Langmuir model) of the tested commercial activated carbons, in the case of the Cr(III) ions, ranged from 55.85 mg/g (F-300) to 63.49 mg/g (ROW 08 Supra) and, in the case of Ni(II), from 40.29 mg/g (F-300) to 51.70 mg/g (WG-12). The adsorption capacities presented above were calculated using the Langmuir model because it is the model most often used by other researchers. However, in many cases higher correlation coefficients were obtained for the Toth, Temkin and Jovanovic models. The lowest R^2^ values were obtained for the Halsey model, which is the only one that assumes multilayer sorption.

Comparing the tested activated carbons, it can be seen that the F-300 sorbent absorbs the smallest amounts of both Cr(III) and Ni(II) ions. The largest amounts of Ni(II) are adsorbed by the activated carbon WG-12, which has the largest amount of acidic surface oxides and the largest amount of carboxylic oxides, which are considered by many authors to be responsible for the adsorption of these ions. In the case of the adsorption of Cr(III) ions, the ROW 08 Supra is the most effective carbon. This carbon does not contain large amounts of carboxylic or lactone oxides, which, according to many authors, are responsible for the sorption of chromium cations. This carbon is characterized by the largest number of mesopores, while F-300, which adsorbs the fewest Cr(III) ions, has the fewest mesopores. The porous structure is one of the parameters which determines the magnitude of adsorption. Moreover, the precipitation mechanism of chromium hydroxide is also an important parameter for adsorption on the ROW 08 Supra activated carbon.

The adsorption kinetics was also analyzed and four models were used to describe it: the PSO, PFO, Elovich and intraparticle diffusion models. The PFO and PSO models are most often used to describe adsorption dynamics, despite the fact that they are semi-empirical in nature. The adsorption kinetics of both the Cr(III) and Ni(II) ions is best described using the PSO model.

Based on the IPD model, the two slowest phases of the process of adsorption of Cr(III) and Ni(II) were determined. For the tested activated carbons, the third stage (intramolecular diffusion) is much slower than the second stage (film diffusion). Diffusion in the pores (k_p2_ < k_p1_) controls the adsorption of the tested metal ions, but it is not the only rate-limiting step during adsorption (the curves q_t_ and t^1/2^ do not pass through the origin of the coordinate system).

Based on the obtained R_L_ values from the Langmuir isotherm, it can be concluded that the adsorption of Cr(III) and Ni(II) ions is efficient for all the commercial activated carbons used in this study.

The adsorption of nickel and chromium ions also occurs in large amounts when the process is carried out using natural water. Even when competing cations (Ca^2+^, Mg^2+^, Na^+^ and K^+^) are present in an aqueous solution at much higher concentrations, the adsorption efficiency decreases by only a few percent. Only in the case of water with a very high degree of mineralization (total ion concentration of 1836.80 mg/L) did the adsorption efficiency decrease by several percent (11, 13%).

## Figures and Tables

**Table 3:** Constants of Cr(III) adsorption isotherms on commercial activated carbons.

Isotherm Model	Activated Carbon	Constants of the Isotherm Model			Model Accuracy Parameters	
Langmuir $q=\frac{q_{m}K_{L}C_{e}}{1+K_{L}C_{e}}$ $R_{L}=\frac{1}{1+\frac{K_{L}}{\;q_{m}}C_{0}}$		q_m_, mg/g	K_L_, L/mg	R_L_^1^	R^2^	E, %
	WG-12	60.73	0.08	0.88–0.97	0.997	1.42
	ROW 08	63.49	0.129	0.83–0.98	0.988	3.44
	F-300	55.85	0.106	0.84–0.98	0.996	1.98
Freundlich $q=\frac{q_{m}K_{L}C_{e}}{1+K_{L}C_{e}}$		1/n, -	K_F_, mg/g	-	R^2^	E, %
	WG-12	0.341	12.58		0.929	9.97
	ROW 08	0.279	18.32		0.863	12.78
	F-300	0.298	14.41		0.907	10.24
Temkin $q=BlnA_{T}C$		A, L/mg	B, -		R^2^	E, %
	WG-12	0.817	13.026		0.999	0.31
	ROW 08	1.547	12.697		0.995	0.59
	F-300	1.202	11.451		0.998	0,.
Jovanovic $q=q_{\infty }\cdot (1-e^{-K_{j}\cdot C_{e}})$		K_j_, L/mg	q_m_, mg/g		R^2^	E, %
	WG-12	0.071	50.51		0.992	3.14
	ROW 08	0.104	54.94		0.997	2.01
	F-300	0.088	47.65		0.990	3.10
Halsey $q=(K_{h}\cdot C_{e}{)}^{1/n_{h}}$		K_h,_ (mg·g^−1^/mg·L^−1^${)}^{1/n_{h}}$	n, -		R^2^	E, %
	WG-12	192,276	4.196		0.855	14.74
	ROW 08	192,276	4.034		0.854	13.73
	F-300	192,276	4.214		0.874	12.32
Redlich–Peterson $q=\frac{K_{R}\cdot C_{e}}{1+a_{R}\cdot C_{e}^{\beta }}$		K_R_, L/g	a_R_, (L/mg)^β^	β	R^2^	E, %
	WG-12	219,670	17,460	0.659	0.929	9.97
	ROW 08	220,093	12,010	0.721	0.863	12.78
	F-300	219,915	15,263	0.702	0.970	10.24
Toth $q=q_{m}\cdot \frac{b\cdot C_{e}}{(1+(b\cdot C_{e}{)}^{v}{)}^{1/v}}$		q_m_, mg/g	b, mg/g	v, -	R^2^	E, %
	WG-12	68.90	0.092	0.773	0.992	2.94
	ROW 08	57.90	0.097	1.524	0.998	1.35
	F-300	52.42	0.90	1.264	0.999	0.85

q_m_—the maximum adsorption capacity; K_L_—the constant related to the free energy of adsorption; R_L_—separator factor; 1/n—adsorption intensity; K_F—_Freundlich isotherm constant; R^2^—correlation coefficient; A—Tempkin isotherm equilibrium binding constant; B—Tempkin isotherm constant; K_j_—the constant related to the free energy of adsorption; K_h—_the Halsey constant; n_h_—the Halsey constant; K_R_, a_R_ and β—Redlich–Peterson isotherm constants; v—parameter characterizing the heterogeneity of the deposit; b—Toth isotherm constant; R_L_—equilibrium parameter. ^1^ R_L_ is not a constant of the Langmuir isotherm, but is calculated from this isotherm.

**Table 6:** Constants of Ni(II) adsorption kinetics equations: the pseudo-first-order, pseudo-second-order, intraparticle diffusion (Weber–Morris) and Elovich models.

Parameter		WG-12	ROW	F-300
Pseudo-first order	q_max_ (mg/g)	35.66	37.73	30.96
	k_1_ (h^−1^)	0.986	0.965	0.751
	R^2^	0.967	0.973	0.989
	E, %	3.77	3.67	3.06
Pseudo-second order	q_max_ (mg/g)	40.68	43.16	36.61
	k_2_ (h^−1^)	0.031	0.029	0.024
	R^2^	0.996	0.997	0.996
	E, %	1.13	0.95	1.26
Elovich	α, mg/(g·h)	135	135	60
	β, g/mg	0.129	0.120	0.123
	R^2^	0.969	0.968	0.971
	E, %	3.63	3.88	4.56
Intraparticle diffusion model (Weber–Morris)	k_p1_, mg/(g·h^0.5^)	14.23	16.77	15.18
	C_1_, mg/g	7.37	5.84	1.00
	R^2^	0.976	0.978	0.983
	k_p2_, mg/(g·h^0.5^)	1.98	2.38	2.42
	C_2_, mg/g	31.10	32.10	24.71
	R^2^	0.999	0.881	0.933

**Table 7:** Comparison of Ni(II) adsorption results based on the reports in the literature.

Activated Carbon	Max. q_m_, mg/g	Adsorption Isotherms Equations Tested	The Equations of Adsorption Kinetics Studied	Ref.
Peat	61.27	L > F	PSO > PFO	[55]
From Mespilus germanica Lea	13.08	L > F	PSO > PFO	[15]
From Citrus limetta leaves	58.14	L > F	PSO > PFO	[56]
Polystyrene based	40.82	L > F	PSO > IPD > Elovich > PFO	[57]
Modified AC	56.18	L > T > F > D–R	PSO > PFO > Elovich	[58]
From biomass	62.9	L > F > D–R	PSO	[59]
From palm kernel chaff	115.7	L ≈ T ≈ D–R > F	PSO > PFO	[60]
From date stones	16.12	L ≥ F	-	[61]
From arundo donax	8.61	F > L	PSO > PFO > IPD	[62]

**Table 8:** Constants of Ni(II) adsorption isotherms on commercial activated carbons.

Isotherm Model	Activated Carbon	Constants of the Isotherm Model			Model Accuracy Parameters	
Langmuir		q_m_, mg/g	K_L_, L/mg	R_L_	R^2^	E, %
	WG-12	51.70	0.067	0.88–0.99	0.998	1.15
	ROW 08	47.48	0.104	0.82–0.98	0.986	3.44
	F-300	40.29	0.082	0.83–0.98	0.987	3.58
Freundlich		1/n, -	K_F_, mg/g	-	R^2^	E, %
	WG-12	0.361	9.497		0.960	7.25
	ROW 08	0.286	12.74		0.869	11.14
	F-300	0.312	9.30		0.888	10.68
Temkin		A, L/mg	B, -		R^2^	E, %
	WG-12	0.677	11.21		0.999	0.13
	ROW 08	1.189	9.71		0.996	0.54
	F-300	0.838	8.59		0.997	0.49
Jovanovic		K_j_, L/mg	q_m_, mg/g		R^2^	E, %
	WG-12	0.061	42.45		0.978	5.04
	ROW 08	0.087	40.61		0.997	1.42
	F-300	0.071	33.82		0.997	1.65
Halsey		K_h,_ (mg·g^−1^/mg·L^−1^${)}^{1/n_{h}}$	n, -		R^2^	E, %
	WG-12	192,276	4.43		0.841	14.37
	ROW 08	192,276	4.41		0.836	13.22
	F-300	192,276	4.69		0.808	14.00
Redlich–Peterson		K_R_, L/g	a_R_, (L/mg)^β^	β	R^2^	E, %
	WG-12	219,372	23,097	0.639	0.960	7.25
	ROW 08	219,986	17,262	0.714	0.869	11.14
	F-300	219,489	23,600	0.688	0.888	10.68
Toth		q_m_, mg/g	b, mg/g	v, -	R^2^	E, %
	WG-12	54.67	0.073	0.878	0.999	1.01
	ROW 08	42.56	0.077	1.627	0.999	0.52
	F-300	35.64	0.062	1.632	0.998	1.39

**Table 9:** Removal efficiency of chromium and nickel ions from natural waters on WG-12 activated carbon, C_0_ =10 mg/L.

Water	Water Composition, mg/L								Adsorption Efficiency, %	
	Anions				Cations					
	$\mathrm{HCO}{}_{3}^{-}$	$\mathrm{SO}{}_{4}^{2-}$	F^−^	Cl^−^	Ca^2+^	Mg^2+^	Na^+^	K^+^	Cr(III)	Ni(II)
Demineralized	-	-	-	-	-	-	-	-	61.5 ± 0.7	54.2 ± 1.0
Spring water (A)	131.06	-	0.07	-	41.69	5.62	9.65	-	60.4 ± 2.3	53.9 ± 0.8
Spring water (B)	180.90	14.82	0.12	3.19	46.09	8.51	6.25	2.00	59.7 ± 1.2	53.5 ± 1.1
Spring water (C)	216.60	10.90	-	4.96	71.14	5.47	1.87	0.50	58.6 ± 2.5	52.3 ± 1.4
Spring water (D)	168.00	14.71	0.09	2.80	50.10	6.08	2.50	1.19	58.3 ± 2.4	52.1 ± 2.3
Low mineralized (E)	186.70	43.62	-	3.19	44.09	17.01	11.10	1.00	57.7 ± 2.8	51.9 ± 2.1
Medium mineralized (E)	299.00	41.60	0.23	-	91.18	16.52	6.44	1.21	56.3 ± 1.8	50.7 ± 2.5
Medium mineralized (F)	432.7	-	0.23	2.50	102.2	16.00	11.25	2.34	55.8 ± 2.1	50.3 ± 2.6
Highly mineralized (G)	1403.7	32.0	-	7.0	180.9	142.7	63.0	7.5	48.3 ± 3.5	43.0 ± 3.2

**Table 10:** SEM/EDS analysis results.

Spectrum	C	O	Na	Mg	Al	Si	S	K	Ca	Cr	Fe	Ni
F-300	66.32	29.92	-	-	1.18	1.14	0.85	-	-	-	0.59	-
	63.72	31.70	-	-	1.53	1.51	0.73	-	-	-	0.80	-
	66.15	29.98	-	-	1.24	1.24	0.83	-	-	-	0.56	-
ROW 08 Supra	70.10	26.00	-	-	0.57	0.98	0.80	-	0.87	-	0.74	-
	72.68	24.66	-	-	0.34	0.44	0.75	-	0.56	-	0.55	-
	72.20	25.15	-	-	0.33	0.52	0.75	-	0.52	-	0.52	-
WG-12	51.13	38.88	-	-	0.90	6.70	0.20	0.54	-	-	1.08	-
	45.75	42.56	-	-	3.40	4.89	0.27	0.50	-	-	1.83	-
	48.63	40.88	-	-	2.88	4.18	0.27	0.56	-	-	1.78	-

**Table 11:** Conditions for the adsorption process.

Process	Adsorption Time, h	Initial Concentration, mg/L	Sample Volume, L	pH of the Solution	Activated Carbon Dose, g/L
Adsorption kinetics	0.5, 1, 2, 3, 4, 5, 6, 7 and 8	50	0.250	6 ± 0.2	0.4
Statics of adsorption	6	10, 20, 30, 40, 50, 60, 80 and 100	0.250	6 ± 0.2	0.4
Influence of adsorption dose	6	100	0.250	6 ± 0.2	0.5, 1, 2, 3, 4, 5 and 6

## Data Availability

Not applicable.
